# Identification, Isolation and Expansion of Myoendothelial Cells Involved in Leech Muscle Regeneration

**DOI:** 10.1371/journal.pone.0007652

**Published:** 2009-10-30

**Authors:** Annalisa Grimaldi, Serena Banfi, Laura Gerosa, Gianluca Tettamanti, Douglas M. Noonan, Roberto Valvassori, Magda de Eguileor

**Affiliations:** 1 Department of Biotechnology and Molecular Sciences, University of Insubria, Varese, Italy; 2 Department of Clinical and Biological Sciences, University of Insubria, Varese and Istituto di Ricovero e Cura a Carattere Scientifico (IRCCS) Multimedica, Milan, Italy; University of Birmingham, United Kingdom

## Abstract

Adult skeletal muscle in vertebrates contains myoendothelial cells that express both myogenic and endothelial markers, and which are able to differentiate into myogenic cells to contribute to muscle regeneration. In spite of intensive research efforts, numerous questions remain regarding the role of cytokine signalling on myoendothelial cell differentiation and muscle regeneration. Here we used *Hirudo medicinalis* (Annelid, leech) as an emerging new model to study myoendothelial cells and muscle regeneration. Although the leech has relative anatomical simplicity, it shows a striking similarity with vertebrate responses and is a reliable model for studying a variety of basic events, such as tissue repair. Double immunohistochemical analysis were used to characterize myoendothelial cells in leeches and, by injecting *in vivo* the matrigel biopolymer supplemented with the cytokine Vascular Endothelial Growth Factor (VEGF), we were able to isolate this specific cell population expressing myogenic and endothelial markers. We then evaluated the effect of VEGF on these cells *in vitro*. Our data indicate that, similar to that proposed for vertebrates, myoendothelial cells of the leech directly participate in myogenesis both *in vivo* and *in vitro*, and that VEGF secretion is involved in the recruitment and expansion of these muscle progenitor cells.

## Introduction

Adult stem cells from tissues such as muscle, bone marrow, or the brain, exhibit the ability to differentiate into diverse cell types according to the cues provided by the tissue in which they have been reintroduced [1], [2]. For example, hematopoietic stem cells (HSCs) can not only produce all blood cell lineages, but also exhibit developmental plasticity, differentiating, for example, into cardiac muscle or vascular endothelium [3], [4] with appropriate stimuli. Myogenic and endothelial (myoendothelial) cell progenitors have been identified in the interstitial spaces of murine skeletal muscle. These primitive cells, which are distinct from satellite cells, are located outside of the basal lamina, express both endothelial and myogenic cell markers and are able to differentiate into myogenic cells, contributing to new fibre formation [5]–[7]. Adult muscle tissue therefore appears to contain a common type of pluripotent stem cells, such as hematopoietic and mesenchymal-like cells [8]. These cells are activated during tissue repair, differentiating in a context–specific manner, presumably in response to growth factors and signals provided by the host tissue [2].

Among the cytokines produced by different tissues, VEGF (Vascular Endothelial Growth Factor) mediates its biological effects by binding to a series of transmembrane tyrosine kinase receptors, principally VEGFR-1 and VEGFR-2 [9]. It plays roles as a potent proangiogenic molecule, as a mitogen and survival factor or as a chemoattractant for endothelial cells [10]–[12] under physiological and pathological conditions. Moreover, it has been demonstrated that VEGF_165_ (the most abundant and biologically active isoform) is a potent chemoattractant for CD34 positive cells and it is critical for the migration of CD34^+^ stem cells from the bone marrow into tumors [13].

Despite extensive investigation and characterization of stem cells showing molecular and developmental relationships between endothelial and myogenic cells in vertebrates, no reports have been published that explore this phenomenon in invertebrates.

We have previously demonstrated that in the leech *Hirudo medicinalis*, the angiogenic process is regulated by several of the principal growth factors and receptors (in particular VEGF and the VEGFRs) that coordinate neovascularization in vertebrates [14], [15]. Moreover, a localized and continuous exposure to VEGF by implantation of a VEGF-Hydron pellet within the muscle wall [15] induced a massive stimulation of circulating cells, as previously described for conditions producing elevated VEGF levels in humans [16]. In *H. medicinalis*, these cells were CD34^+^, and were localized in the vessel lumen as well as disseminated into the extracellular matrix (ECM) [17]. These cells were able to differentiate, *in situ*, into endothelial cells in a manner that reflects that of murine circulating endothelial progenitor cells [18]. Interestingly, CD34^+^/Flt-1^+^/CD45^−^ endothelial cells isolated from skeletal muscle have also been found to differentiate into skeletal muscle fibres [19].

During leech wound healing, new vessels are formed in otherwise avascular tissues. The botryoidal tissue is composed of two different cell types, granular botryoidal cells and flattened endothelial-like cells that are organized in cords or clusters, sometimes surrounding occasional small lacunae localized in the loose connective tissue between the gut and the body wall sac. In wounded animals, the botryoidal tissue undergoes a transition from cluster/cord-like structures to a hollow, tubular architecture typical of pre-vascular structures that gives rise to new capillary vessels [17]. Concurrently, groups of small, closely-associated cells became evident in the centre of the immature vessel lumen. They are initially adherent to the vessel wall and appear to be precursor cells. As the vessel grows, these circulating precursor cells lose cell-cell contacts to move freely within the lumen. Most of the circulating precursor cells are channelled into the wounded area via the new vessels, where they extravasate and disperse into the connective tissue. These circulating precursor cells in the leech express many of the same markers as those commonly used to identify vertebrate ECs and hematopoietic stem cells [17].

Here we examined whether in leeches, like in vertebrates, the vessel-associated precursors cells, dispersed in the connective tissue of the body wall muscle, are responsible for muscle regeneration during wound healing repair. Our data show that in the leech a population of cells, showing features in common with myoendothelial cells found in vertebrate skeletal muscle [6], [7], are dispersed in the ECM surrounding the muscle fibres. These cells coexpress both myogenic (MyoD and desmin) and endothelial markers (Flk-1, VE-cadherin, CD34 and CD31) and are recruited by the growth factor VEGF into the area undergoing regeneration. This specific population of cells was isolated and cultured by using the biopolymer matrigel supplemented with VEGF [20] and its differentiation in muscle cells was assessed *in vivo* and in *vitro*.

## Results

### Identification of Leech Myoendothelial Precursors Cells

#### Morphological, immunohistochemical and biochemical analysis

**Figure 1:**
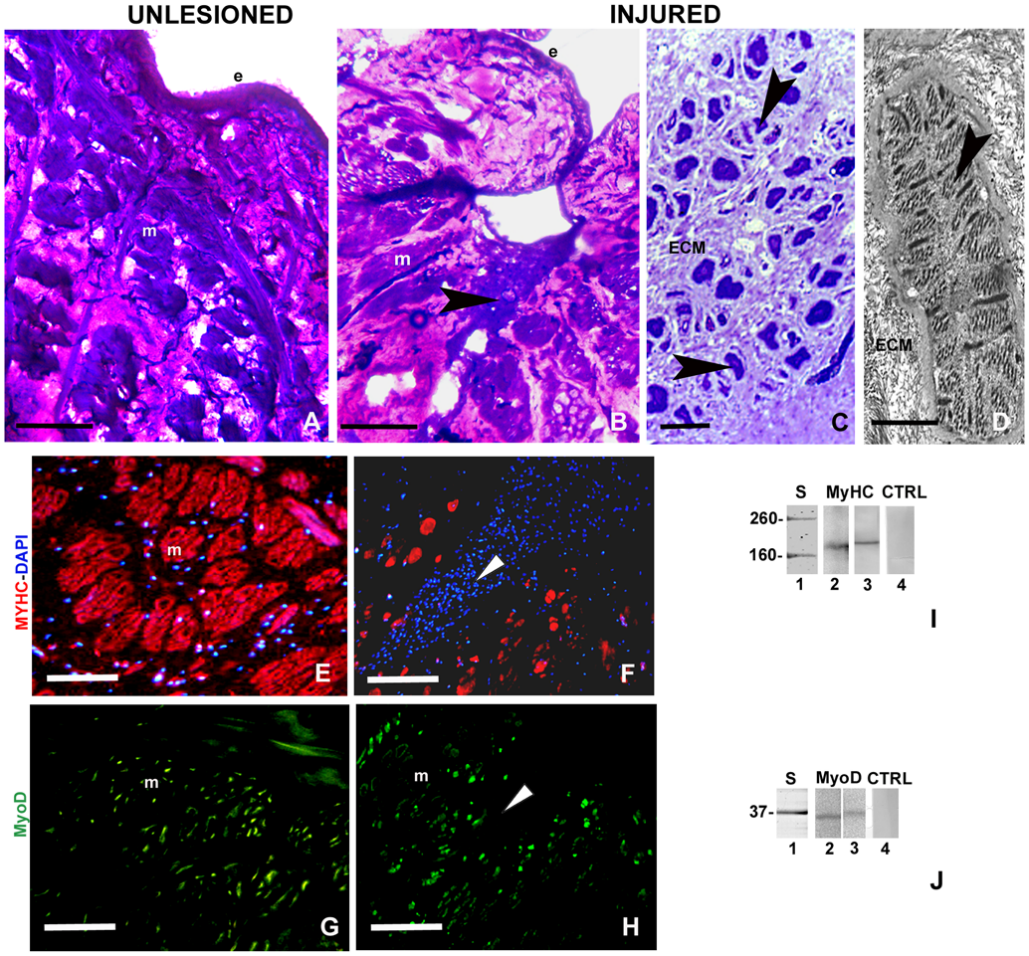
Morphological, immunoistochemical and biochemical assays of unlesioned (A, E, G) and injured (B–D, F, H) leech muscle body wall. (A) Cross cryosection of the body wall of an unlesioned leech. Under the epithelium muscular layers, circularly, obliquely and longitudinally oriented are visible. (B) Cross cryosection of the body wall of a leech one week after a surgical lesion. A plug (arrowhead) formed by migrating cells is visible under the epithelium. (C, D) Detail at the optical (C) and electron (D) microscope level of the regenerating area after one month from injury. Small muscle cells (arrowhead in C), with their contractile material organized in sarcomeres (arrowhead in D) are surrounded by an abundant collagen matrix (ECM in C and D). (E–H) The muscle cells are stained by MyHC (in red) and MyoD (in green) antibodies. Nuclei are counterstained with DAPI (blue in E, F). e: epithelium; m: muscle; arrowhead: plug region. (I, J): Western blot analysis. Expression of MyHC A4.1025 (I) and MyoD (J) in extracts of unlesioned *H. medicinalis* body walls or rat muscle used as positive control. The antibody A4.1025 anti-MyHC detects a band of about 200 kDa both in rat (I, lane 2) and in leech (I, lane 3). The antibody anti-MyoD recognizes the expected band of about 34 kDa in rat (J, lane2) and a band of 37 kDa in leech (J, lane 3). The molecular weights are in accordance with those of the corresponding vertebrate molecules. Lanes 1 in I, J: molecular weight standards. Lanes 4 in I, J: negative controls. Bars in A, B, E–H: 100 µm; Bar in C: 10 µm; Bar in D: 5 µm.

The unlesioned leech body wall (Fig. 1A) is essentially made up of an epithelium wrapping grouped muscle fibres embedded in a loose connective tissue. Following tissue damage, wound healing initiates with an inflammatory phase [21], [22] characterised by a massive migration of macrophages and fibroblasts (Fig. 1B), followed by fibroplasia and angiogenesis, with the formation of a network of blood vessels spanning the entire body wall [17]. Approximately 1 month after injury, small new muscle fibres are visible in the abundant newly synthesised collagen matrix (Fig. 1C, D). Based on these observations, we examined whether myoendothelial cells, of both endothelial and myogenic origin, could be involved in muscle regeneration in leeches, similar to that observed in vertebrates [6], [7], [23]. Sections of both unlesioned and injured leeches were immunostained with antibodies against antigens characteristic of myogenic, endothelial or macrophages cells. Muscle cells expressed the myosin heavy chain (MyHC) (Fig. 1E, F) and the muscle regulatory factor (MRF) MyoD (Fig. 1G, H) both in control and injured animals. To confirm the specificity of antigen recognition by the anti-MyoD and anti-MyHC, antibodies, Western blot analyses were performed to compare a protein extract from the body muscles of leeches with extracts from rat muscle used as a positive control. The anti-MyHC A4.1025 antibody revealed a band of 200 KDa both in rat and in leech samples (Fig. 1I lanes 2 and 3, respectively). The anti-MyoD antibody detected a band of the expected 34 kDa size in rat (Fig. 1J, lane 2) and a band of approximately 37 kDa in leech (Fig. 1J, lane 3). Omission of the primary antibodies did not stain any proteins in the leech extracts (Fig. 1I and 1J lanes 4).

**Figure 2:**
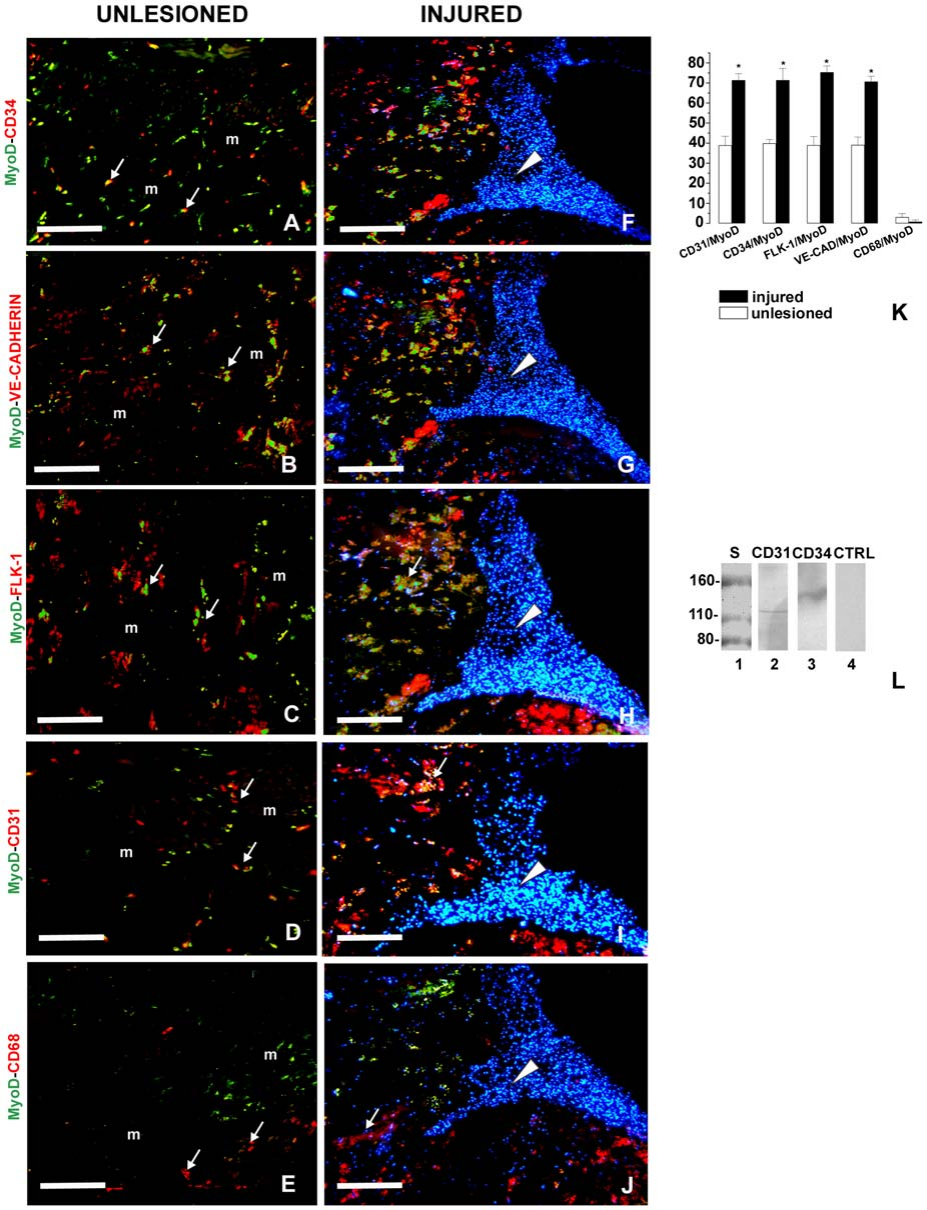
Double immunolocalization of endothelial and myogenic markers in the interstitial spaces of leech skeletal muscle. (A–E) Cryosections of unlesioned leech. (F–J) Serial cryosection of injured leeches. Small rounded cells (arrows) dispersed among muscle (m) in control animals (A–E) and close to the regenerating area (arrowheads) in wounded leeches (F–J) express the MyoD muscle transcriptional factor (green) and the hematopoietic and endothelial markers CD34 (in red A, F), VE-cadherin (in red B, G), Flk-1 (in red C, H), CD31 (in red D, I). CD68^+^ cells (in red E, J) are MyoD^−^ (in green E, J) and in injured animals (J) are mainly located in the regenerating area (arrowhead). The muscle cells (m) do not express the endothelial or the macrophage markers (A–E). (K) The percentage of cells expressing myogenic, endothelial and macrophage markers. (L) Expression of CD markers in injured leeches. The antibodies detected a band of 120 kDa for CD31 (lane 2), 140 kDa for CD34 (lane 3). The molecular weights are in accordance with those of the corresponding vertebrate molecules. Lanes 1 molecular weight standards. Lanes 4: negative controls. Bars in A–J: 100 µm.

Cells expressing the typical endothelial cell markers CD34, VE-cadherin, Flk-1 and CD31 [6], [7], [15], [17], [20] and the macrophage cell marker CD68 [17], [20], [24] were found dispersed in the ECM surrounding the groups of muscle cells in the control unlesioned animals (Fig. 2A–E) and close to the wound healing region of injured leeches (Fig. 2F–J). We never observed the expression of these markers in leech muscle, as clearly visible in unlesioned animals (Fig. 2A–F) or in other leech tissues such as gut (data not shown). Some of the CD34^+^ (Fig. 2A, F), VE-cadherin^+^ (Fig. 2B, G), Flk-1^+^ (Fig. 2C, H) and CD31^+^ (Fig. 2D, I) were also MyoD^+^. In contrast, the CD68^+^ cells, mainly localized in the lesioned/regenerating area, were MyoD^−^ (Fig. 2E, J). Cells that were CD31^+^/MyoD^+^ were 38.9±4.4% of the CD31^+^ cells, CD34^+^/MyoD^+^ 39.8±2% of the CD34^+^ cells, VE-cadherin^+^/MyoD^+^ 39±4% of the VE-cadherin^+^ cells and Flk-1^+^/MyoD^+^ 38.9±4.3% of the Flk-1^+^ cells in unlesioned leeches, while the percentage of these cells rose to 71.3±3%, 71.4±6%, 70.7±3% and 75.3±3.1% respectively, in injured animals (p<0.01, n = 4). The CD68^+^/MyoD^+^ cells were 3±0.2% in unlesioned and 0.8±0.8% of the CD68^+^ cells (the differences were not statistically significant, n = 4) in wounded leeches (Fig. 2K).

The antibodies against the endothelial markers CD31 and CD34 in protein extracts of leech tissue stained proteins of approximately 120 kDa for CD31 (Fig. 2L, lane 2) and 140 kDa for CD34 (Fig. 2L, lane 3). These molecular weights correspond to those of the murine counterparts. Omission of the primary antibodies did not stain any proteins in the leech extracts (Fig. 2L, lanes 5). Confirmation of antibody specificity by western blotting for CD68, Ve-cadherin and Flk-1 have been previously published [15], [17], [25].

#### Dil-Ac-LDL up-take

**Figure 3:**
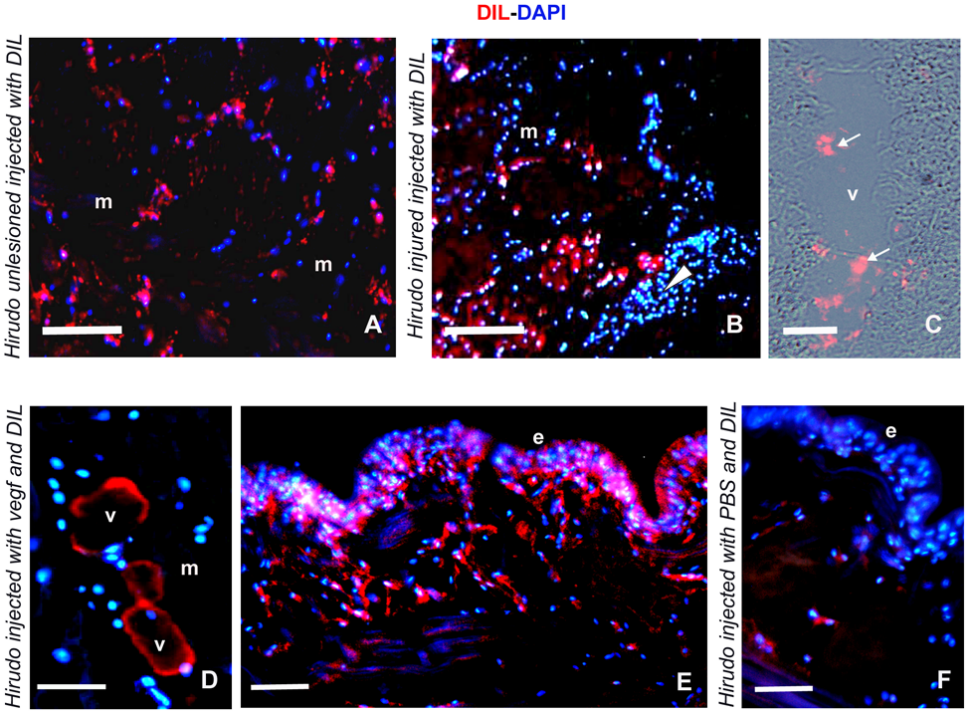
Dil-Ac-LDL uptake by myoendothelial cells located in the interstitial spaces of leech skeletal muscle and recruited by VEGF injection. (A–C) Cryosections of unlesioned (A) and injured (B, C) leeches injected with Dil-Ac-LDL. Samples were processed 1 week after treatment. Dil^+^ cells (red) are visible dispersed in the ECM surrounding the groups of muscle fibres (m) in control animal (A), close to the regenerating area (arrowhead in B) and in the lumen of vessels (v in C) in the injured leeches. (D–F) Cryosections of a VEGF/Dil-Ac-LDL injected leech. After one week from injection new vessels (D) and cells incorporating the vital colorant Dil-Ac-LDL (red in D, E) are visible under the epithelium (e) in the injected area. In control PBS/DIL injected areas, few Dil^+^ cells (red in F) are present under the epithelium (e). Nuclei are counterstained with DAPI (blue in A, B, D–F). Bars in A, B: 100 µm; Bar in D–F: 50 µm.

In vertebrates, co-expression of endothelial and myogenic markers in combination with acetylated low-density lipoporotein (Ac-LDL) conjugated with the fluorochrome probe Dil (Dil-Ac-LDL) uptake is associated with a population of myoendothelial cells located in the muscular interstitial spaces and involved in muscle regeneration [6], [7]. We carried out experiments to establish whether also in leeches a specific population of myoendothelial cells could be involved in leech muscle regeneration. To address this, Dil-Ac-LDL (which is taken up preferentially by endothelial cells and macrophages via the “scavenger cell pathway” of LDL metabolism [6], [7], [26]) was used as a vital marker to stain and to follow the fate of those cells interspersed in the interstitial space of muscles in unlesioned leeches, or localized close to the injured region in wounded leeches. After 4 h from injection of Dil-Ac-LDL in both the control healthy and in the injured leeches, cryosections of unlesioned (Fig. 3A) and injured animals (Fig. 3B, C) were analysed. Numerous cells dispersed among the groups of muscle cells in unlesioned leech (Fig. 3A) or localised in the injured, regenerating area of the muscular sac (Fig. 3B) showed uptake of the fluorochrome-conjugated probe Dil-Ac-LDL, indicating scavenger receptor expression typical of endothelial cells and macrophages. DIL-Ac-LDL uptake was also detected in the cytoplasm of many circulating precursors cells found within the lumen of newly formed vessels (Fig. 3C). Notably, mature muscle cells did not take up Ac-LDL, (Fig. 3A, B).

### VEGF Is Involved in the Recruitment of Dil-Ac-LDL^+^ Cells

We previously reported that VEGF_165_ in the leech promoted not only angiogenesis and recruitment of hematopoietic precursor cells [15] but also the migration of Dil-, CD34^+^ cells into injected matrigel biopolymer supplemented with VEGF [15], [20]. Since in vertebrates the CD34 is a marker associated not only with hematopoietic precursors/endothelial cells, but also with the population of myoendothelial cells involved in muscle regeneration [6], [7], we sought to determine whether in the leech a similar population of myoendothelial cells, recruited by VEGF, could be involved in leech muscle regeneration. We first assessed the ability of VEGF to influence mobilization of the Dil-Ac-LDL^+^ cells *in vivo*. Consistent with this, angiogenesis and migration of cells was stimulated by VEGF injection in the body wall muscle of leeches that had been previously injected with Dil-Ac-LDL. One week after VEGF and Dil-Ac-LDL injections, numerous vessels (Fig. 3D) and cells incorporating Dil-Ac-LDL (Fig. 3E) were clearly visible in the region corresponding to the area under the epithelium of the injection site. Few Dil^+^ cells were observed in PBS/Dil injected control animals (Fig. 3F).

### Isolation, Characterization and Culture of Cells Dispersed in the Interstial Muscle Space

**Figure 4:**
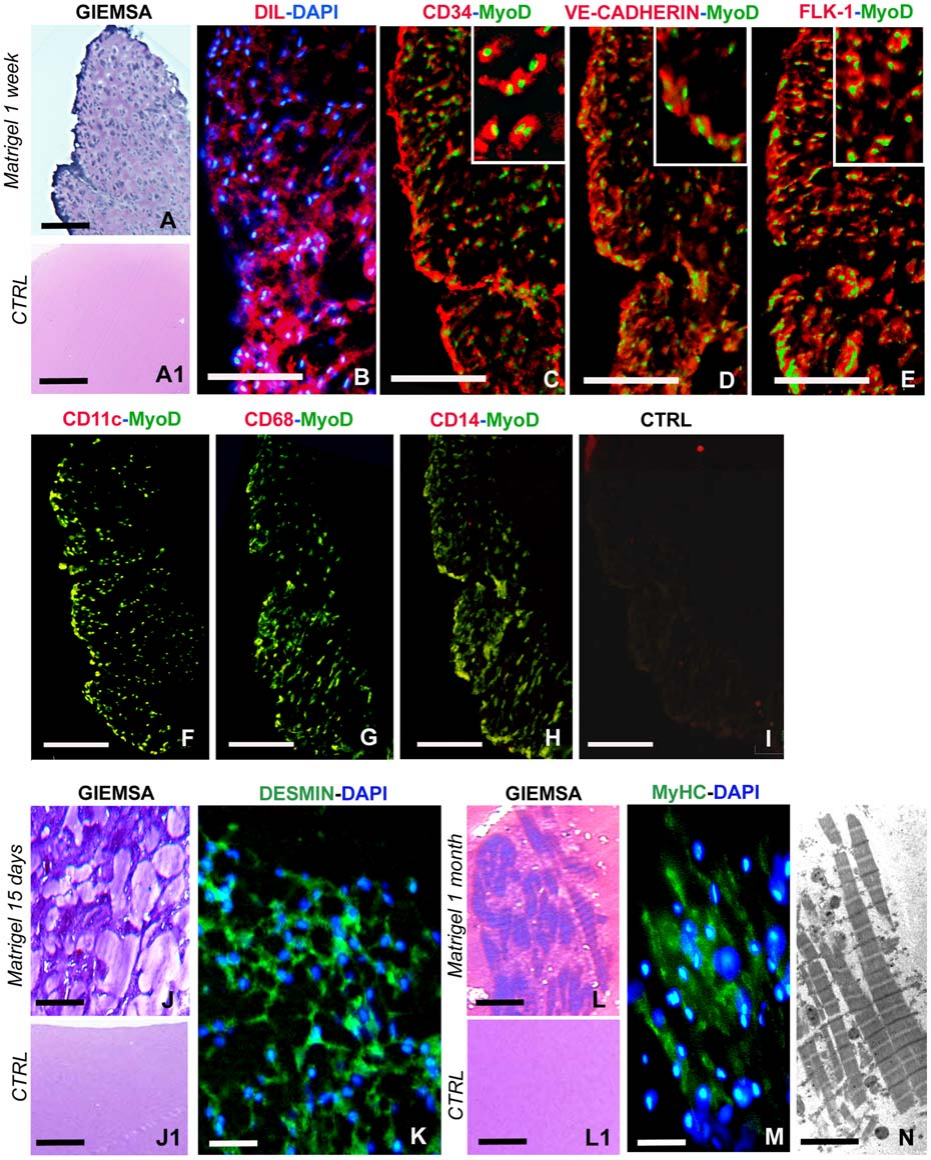
Characterization of myoendothelial cells recruited into the matrigel sponges by VEGF. (A, B) Cryosections of MG, supplemented with VEGF and inoculated in leeches after an intramuscular Dil-Ac-LDL injection. After 1 week *in vivo* the matrigel implant contains rounded cells stained with Giemsa (A) and showing uptake of Dil-Ac-LDL (red in B). (C–I) Double labelling on serial cryosections of MG, supplemented with VEGF and inoculated in leeches. After 1 week *in vivo*, MG is infiltrated by cells expressing the MyoD muscle transcriptional factor (green in C–E and in the inserts) and the hematopoietic/endothelial precursor markers either CD34 (red in C and the insert), VE-cadherin (red in D and the insert), or Flk-1 (red in E and the insert). No staining is detected for the macrophage markers CD11c (red in F) CD68 (red in G) and CD14 (red in H). (I) Negative control where the primary antibodies were omitted and the secondary antibodies were applied together. After 15 days (J, K), MG is infiltrated by elongated Giemsa stained cells (J) that expressed desmin (green in K). After 1 month (L–N) groups of differentiated muscle fibres (L) are present in the MG sponge that are stained by the anti-skeletal MyHC A4.1025 antibody (green in M) and show the contractile material organized in sarcomeres with TEM (N). Nuclei are counterstained with DAPI (blue in B, K, M). (A1, J1, L1): cryosections of matrigel without VEGF injected as controls. Bars in A, A1, F-1: 100 µm; Bars in B–E: 50 µm; J, J1, K: 20 µm; Bars in L, L1, M: 10 µm; Bar in N: 2 µm.

In order to follow the fate of Dil-Ac-LDL^+^ cells and to assess their potential to differentiate into myogenic cells *in vivo* and *in vitro*, we used the biopolymer matrigel supplemented with VEGF as a method for isolating these cells *in vivo* and *in vitro* [20]. DIL-Ac-LDL was injected intramuscularly, followed by VEGF supplemented matrigel. The biopolymer, removed after one week, was sparsely colonized by rounded, agranular cells morphologically similar to vessel-associated precursor cells, as previously described [20]. These cells were positive for differential May Grunwald Giemsa staining (Fig. 4A) and took up Dil-Ac-LDL (Fig. 4B).

Once demonstrated that Dil-Ac-LDL^+^ cells dispersed in the matrigel biopolymer derived from the muscular interstitial space, we performed a similar experiment, injecting only matrigel loaded with VEGF without the previous intramuscular Dil injection. The matrigel was then removed from the leech after one week, 15 days or one month from injection and sections of the biopolymer were immunostained with antibodies to endothelial, myogenic and macrophage cell markers. The cells infiltrating the matrigel, removed after one week, expressed the myogenic marker MyoD along with the endothelial markers either CD34, VE-cadherin or Flk-1 [7], as demonstrated by double labelling experiments (CD34/MyoD, VE-cadherin/MyoD, Flk-1/MyoD) on serial sections (Fig. 4C–E). Further, these cells did not express the macrophage markers CD11C [27], CD68 [24] or CD14 [28], whose reactivity with leech macrophages has been previously demonstrated (see methods) (Fig. 4F–H). Thus the MyoD^+^ and endothelial markers positive cells did not show a myeloid phenotype. No staining was detected in negative control experiments (Fig. 4I).

Starting from day 15 *post* MG injection (Fig. 4J–K), the infiltrated cells exhibited a differentiating phenotype. They showed an elongated spindle-like shape (Fig. 4J) and highly expressed desmin (Fig. 4K). Desmin is considered one of the earliest myogenic markers [29], suggesting commitment toward myogenic cell differentiation. One month after MG injection, the infiltrated cells were clearly spindle shaped, showed the typical striations of skeletal muscle cells (Fig. 4L) and expressed the skeletal muscle myosin heavy chain (MyHC) (Fig. 4M). Ultrastructural examination confirmed the presence of contractile material organized in sarcomeres (Fig. 4N). Cellular infiltrates were not observed in injected matrigel polymer that was not supplemented with VEGF (Fig. 4A1, J1, L1).

**Figure 5:**
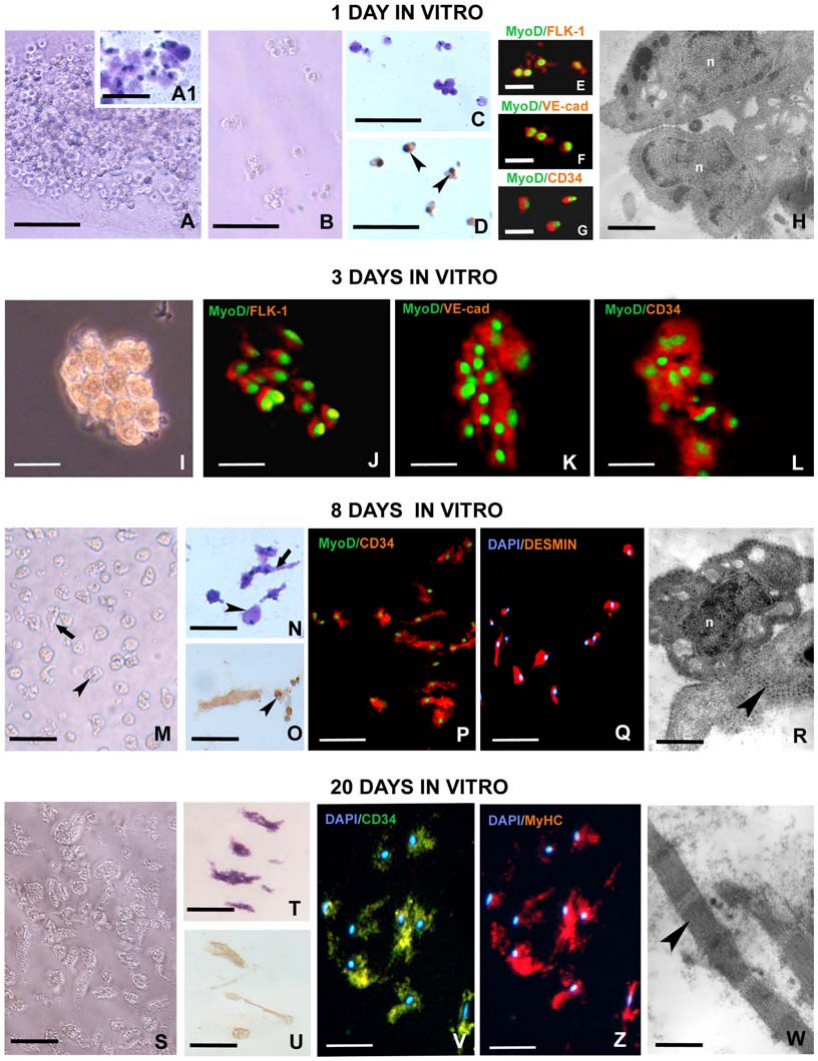
Culture of cells recruited into the matrigel sponges by VEGF. After 1 week *in vivo* the MG was removed and the cells infiltrating the matrigel sponge were plated out at a low density and allowed to form colonies over 24 hrs. (A) Phase contrast image of Matrigel extracted after 1 week *in vivo*. It is infiltrated by numerous rounded cells that stained with Giemsa solution (A1). (B) Phase contrast image of small colonies after 24 hrs from seeding formed by rounded cells which stained with Giemsa (C) incorporate BrdU in their nuclei (arrowheads in D). (E–G) Double labelling experiments showing the expression in these cells of the myogenic marker MyoD (green in E–G) and the endothelial markers FLK-1 (red in E), VE-cadherin (red in F), and CD34 (red in G). (H) TEM micrograph of precursors cells showing an undifferentiated phenotype with large nucleus (n) and scarce cytoplasm. (I) Phase contrast image of a single colony that was picked with a micropipette and clonally expanded for a total of 3 days in vitro. (J–L) Double labelling experiments of clonal colonies showing the expression of MyoD (green in J–L) and the endothelial markers FLK-1 (red in J), VE-cadherin (red in K) or CD34 (red in L). (M) Clonal expansion of single colonies in phase contrast after 8 days in vitro. Rounded (arrowhead) and spindle-shape cells (arrow) that stained with Giemsa solution (N) are visible. (O) Positivity for BrdU incorporation is detectable only in the nuclei (arrowhead) of the rounded cells. Double immunofluorescence experiments detect the expression of CD34 (red in P) and MyoD (green in P) in the cultured cells. These cells are desmin^+^ as well (red in Q). (R) TEM image showing thick filaments (arrowheads) in differentiating elongated cells. Phase contrast image (S) and Giemsa staining (T) of spindle-shape cells that dominate the clonal cultures after 20 days in vitro. The differentiating spindle shaped cells are BrdU^−^ (U), CD34^+^ (green in V) and skeletal MyHC^+^ (red in Z). (W) TEM photomicrograph showing a well differentiated muscle fibre with contractile material clearly organized into sarcomeres (arrowhead in W). Nuclei are counterstained with DAPI (blue in Q, V, Z). Bars in A–D: 100 µm; Bars in E–G: 20 µm Bars in I–L, V, Z 15 µm; Bars in M–Q, S–U: 10 µm; Bars in H, R, W: 400 nm.

To determine the growth characteristics and differentiation of the cells infiltrating the matrigel, these cells were clonally isolated and cultured from the matrigel polymers that had been removed from the leeches after 1 week in *vivo*. The rounded agranular cells that had infiltrated the matrigel sponge in response to VEGF in vivo (Fig. 5A, A1), were plated out at a low density and allowed to form colonies over a 24 hr period. After 1 day (Fig. 5B–H) these cells, grouped in small clones of 1–3 cells, were morphologically homogeneous (Fig. 5B, H) and stained with Giemsa (Fig. 5C). They were proliferating as demonstrated by BrdU positivity (Fig. 5D) and expressed the following associated myogenic and endothelial markers in double labeling experiments: MyoD/Flk-1 (Fig. 5E), MyoD/VE-cadherin (Fig. 5F) and MyoD/CD34 (Fig. 5G). The percentages of cells MyoD^+^/Flk1^+^, MyoD^+^/VE-cadherin^+^, MyoD^+^/CD34^+^ were 79%, 78% and 80%, respectively, while the percentages of cells that were MyoD^−^/Flk1^+^, MyoD^−^/VE-cadherin^+^, MyoD^−^/CD34^+^ were 20%, 21% and 19%, respectively, as determined by counting a mean of 70 cells in four independent experiments. MyoD^+^/Flk1^−^, MyoD^+^/VE-cadherin^−^, MyoD^+^/CD34^−^ cells were not detected. Distinct colonies of 1–3 cells were then aspirated using a micropipette and transferred into 96 well plates to form clonal cultures. After additional 48 hrs (total 3 days of cultures) the colonies were formed by 10–15 cells (Fig. 5I). Double labeling experiments showed that all the cells forming each colony expressed the myogenic and the endothelial markers MyoD/Flk-1, MyoD/VE-cadherin, MyoD/CD34 (Fig. 5J–L). The individual colonies of rounded cells were expanded *in vitro* and after 8 days from seeding (Fig. 5M–R) showed two different phenotypes, both rounded and spindle shaped cells (Fig. 5M). All the cells stained positively with May Grunwald Giemsa (Fig. 5N), expressed the endothelial marker CD34 and the muscle precursor markers MyoD (Fig. 5P) and desmin (Fig. 5Q), while only the rounded cells were proliferating as demonstrated by BrdU incorporation (Fig. 5O). The presence of few myosin filaments in the cytoplasm of the elongating cells (Fig. 5R) was a clear indication that these cells were undergoing a program of myogenic differentiation. After 15–20 days from seeding in cell culture (Fig. 5S–W), although most of the cells showed a highly spindle shaped differentiated phenotype (Fig. 5S, T) and no longer incorporated BrdU (Fig. 5U), they maintained both endothelial and myogenic features. Most of these cells still expressed hematopoietic/endothelial markers such as CD34 (Fig. 5V), and were also positive for the skeletal MyHC (Fig. 5Z). The percentage of cells MyHC^+^/CD34^+^ was 81% as determined by counting a mean of 40 cells in five independent experiments. Transmission electron microscopy demonstrated a differentiated phenotype, showing elongated cells with clearly organized contractile sarcomeres in the cytoplasm (Fig. 5W). These data show that clones derived from single cells or small colonies underwent a differentiation program going from the proliferating myoendothelial phenotype to a quiescent, muscle phenotype.

### Effects of VEGF *In Vitro*

**Figure 6:**
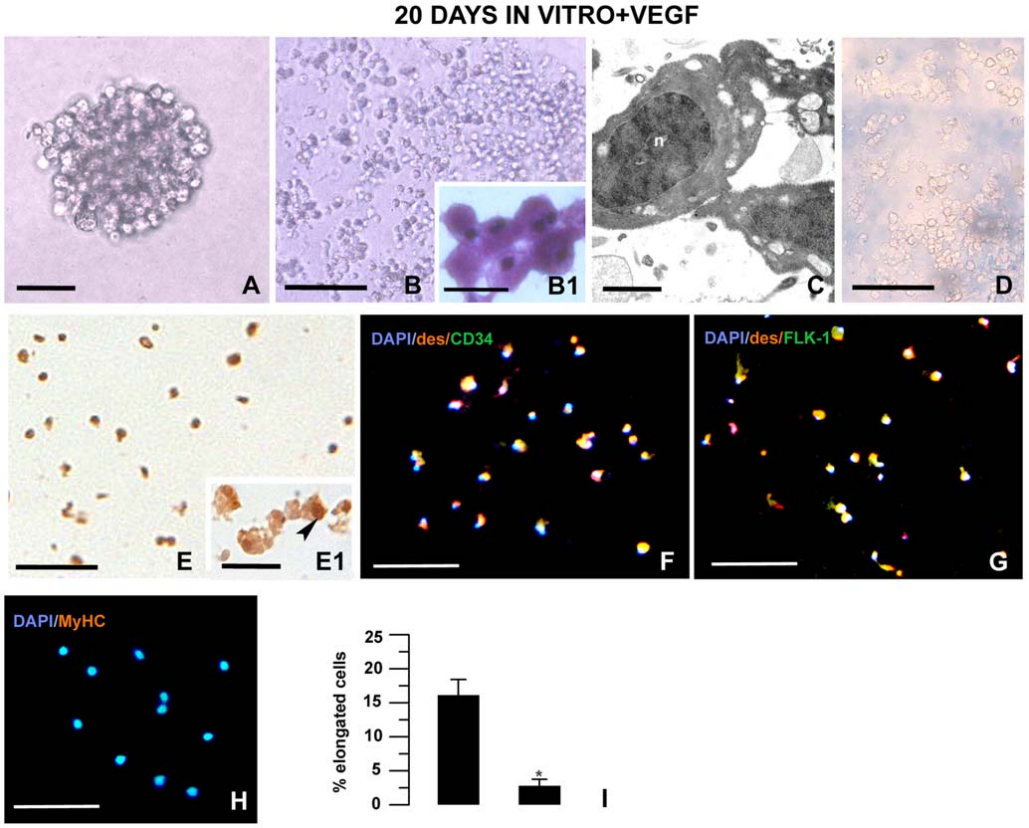
Effect of VEGF on cell differentiation *in vitro*. (A) Phase contrast image of a single colony that was picked with a micropipette and clonally expanded for a total of 3 days in vitro being exposed to VEGF from day 1 (B–H): Photomicrographs were obtained from clonally expanded colonies the were continually exposed to VEGF for a total of 20 days in vitro. (B) Expansion of a single colony in tissue medium. May Grunwald Giemsa staining (B1) and ultrastructural analysis (C) images show that the cultures consisted largely of rounded undifferentiated cells. (D) The Trypan blue assay showing that most of cells are viable (excluding the dye) after 20 days in VEGF supplemented medium (the blue background is due to vital dye precipitation). These cells are proliferating (E) as demonstrated by BrdU incorporation in their nuclei (arrowhead in E1). Double labelling staining showing the expression of desmin (red in F) and CD34 (green in F) or desmin (red in G) and FLK-1 (green in G). The cells exposed to VEGF in vitro for 20 days are MyHC^−^ (red in H). Nuclei are stained with DAPI (blue in F, G, H). (I) Quantitative evaluation of elongated cell numbers. Column 1: cells cultured for 20 days in a VEGF free.medium. Column 2: cells cultured for 20 days in a VEGF supplemented medium.*p<0,01 with respect to the control. Bars in A: 20 µm; Bars in B, D, E, F–H: 100 µm; Bars in B1, E1: 10 µm; Bar in C: 400 nm.

After being picked with a micropipette, some single cell colonies were expanded in medium containing VEGF (Fig. 6A–H) to evaluate the direct effect of this cytokine on the myogenic differentiation program of the leech myoendothelial cells. In the presence of VEGF, these cells maintained their undifferentiated rounded phenotype (Fig. 6A–C), were still viable, as demonstrated by a Trypan blue exclusion (Fig. 6D) and continued proliferating as indicated by BrdU incorporation (Fig. 6E–E1). Although these cells co-expressed desmin and the endothelial markers CD34 (Fig. 6F) or Flk-1, (Fig. 6G), they did not differentiate into muscle fibers nor did they express skeletal MyHC (Fig. 6H).

In four different experiments seeded with identical cell numbers, we found that after 20 days, the growth ratio of VEGF–treated cells was significantly higher than control untreated cells (VEGF, 17.98±1.99 vs control, 8.54±1.97; p<0.05, n = 4). Statistical analysis (Fig. 6I) showed that in the presence of VEGF, the percentage of elongated differentiating cells (as compared to the rounded undifferentiated cells) was significantly lower with respect to cells cultured without VEGF (control 16.06±2.37% vs. VEGF 2.74±1.04%; p<0.01, n = 30 fields).

## Discussion

When leech is subjected to a lesion affecting the entire body wall, a plug constituted by fibroblasts and macrophages is formed [21], [30], [31], [32,]. Fibroblast activation results in synthesis of a high levels of ECM, and during the first days of the healing process, a new robust collagen “scaffold” is produced that directs and regulates cell migration and guides new vessel formation. Once the wound is closed, after 1 month from injury, small new muscle fibres were present in the scarred area and after 2 months the body wall appears completely regenerated [21].

We examined the origin of these new muscle fibres, since in leeches no satellite cells, residing in vertebrate muscles and responsible for regeneration and growth [33], [34], have been detected. Moreover, even if differentiated leech muscle fibres are able to divide in a very peculiar manner [35], this slow process cannot be considered an efficient system for a rapid re-establishment of the muscular body wall.

Like in vertebrates, where the myoendothelial cells are considered an important source of myogenic precursors [6], [7], also in leeches a similar type of cell is present that participates in a rapid regeneration of the lesioned muscle. Using immunohistochemical analysis, we documented the presence of cells expressing the endothelial markers CD34/CD31/VE-cadherin/Flk-1 among the muscle fibres in unlesioned animals. Moreover, double labelling immunohistochemical experiments demonstrated that some of these endothelial cells expressed the myogenic marker MyoD, suggesting they are a source of myogenic precursors similar to the myoendothelial cells identified in vertebrates. Consistent with this concept, only the percentage of endothelial cells coexpressing MyoD, and not endothelial cells that did not express MyoD, increased in the injured animals. Further, the endothelial marker^+^/MyoD^+^ cells were mainly located in the regenerating area. As expected also the percentage of macrophages cells increased in the lesioned area, but these cells were MyoD^−^. Finally, clonal analysis showed that the endothelial marker^+^/MyoD^+^ cells were capable of differentiating into muscle cells *in vitro*.

Endothelial and circulating precursor cells, derived from the leech mesangioblast-like tissue and expressing the hematopietic/endothelial marker CD34 [17] and the VEGF receptor Flk-1 [15], have been previously found dispersed in the interstitial space of leech skeletal muscle. The number of these cells dramatically increased after lesioning or VEGF administration in the leech body wall.

To asses a relationship between vessel-associated circulating precursors and myoendothelial cells, and to follow the fate of these cells during the regenerating process, we used the vital marker Dil-Ac-LDL, which is taken up via the “scavenger cell pathway of LDL metabolism. This marker has been previously used in vertebrates to identify and characterize myoendothelial cells [6], [7], [26]. After Dil-Ac-LDL injection in the leech body wall, DIL^+^ cells were found dispersed within the muscle interstitial space of unlesioned leech, migrated towards the wounded region in the injured leech. A similar effect was obtained by injecting VEGF along with Dil-Ac-LDL in the body wall of leeches, showing numerous vessels and DIL-Ac-LDL^+^ cells migrated into the stimulated region. The same type of cells also migrated into VEGF loaded matrigel biopolymers injected into the leeches. One week after injection, the biopolymer extracted from leeches was invaded by a homogenous type of cells which took up the vital marker Dil-Ac-LDL and selectively expressed CD34/MyoD, CD31/MyoD, VE-cadherin/MyoD, Flk-1/MyoD, but were negative for the macrophage markers CD14, CD11c and CD68, which have been previously used to characterize macrophages in leeches [20]. Fifteen days after injection, the extracted matrigel was highly infiltrated by myocytes expressing desmin and after one month *in vitro*, differentiated muscle fibers expressing the skeletal myosin MyHC were present in the matrigel. Since in leeches the preexisting mature muscle cells were CD34^−^/CD31^−^/VE-cadherin^−^/Flk-1^−^/CD34^−^, we hypothesized that the new differentiated muscle fibres observed in the matrigel biopolymer were derived from the differentiation of myoendothelial cells and not from the migration of already differentiated muscle cells.

To confirm this hypothesis we cultured the precursor cells that had infiltrated the matrigel after 1 week. This approach demonstrated that matrigel can be used to purify a specific population of cells showing the morphological features of vessel-associated precursor cells. We then isolated individual cell colonies from the extracted matrigel and we cultured them *in vitro* for several days and analyzed them morphologically and immunohistochemically. After 24 hrs of culture, most of the proliferating cells forming these colonies expressed the same markers of vertebrate myoendothelial cells [7]. The MyoD^+^/Flk-1^+^, MyoD^+^/VE-cadherin^+^, MyoD^+^/CD34^+^ cells, cultured in a normal medium, spontaneously differentiated into muscle fibres after 15–20 days, while the same type of cells cultured in a medium supplemented with VEGF maintained their undifferentiated phenotype and continued to proliferate.

The main conclusion of this study is that the circulating precursor cells involved in endothelial cell differentiation and contributing to neovascularization during wound healing or after cytokine stimulation [17] can also differentiate into muscle cells and significantly contribute to muscle regeneration in leeches. We hypothesized that the improved myogenic potential of precursors cells is due to their higher proliferation rate, as opposed to the limited proliferative capacity of differentiated muscle fibres.

The apparent different myogenic and endothelial destinies of these precursor cells could be due to differential responses to VEGF concentrations, as suggested previously [15]. These cells can be driven toward a myogenic differentiation pathway, depending on the time course of VEGF released to target cells. When CD34^+^/CD31^+^/Ve-cadherin^+^/Flk-1^+^ cells are exposed to a sustained and continuous source of VEGF, slowly diffusing from injected matrigel or perhaps from physiological ECM “sinks”, they proliferate and principally maintain their undifferentiated phenotype as precursor cells. As the VEGF concentration decreases (inside the matrigel, or in the ECM surrounding the muscle fibres) the proliferation stimulus diminishes, permitting these hematopoietic/endothelial precursors cells to then differentiate into muscle fibres. The secretion of VEGF during wound healing could be interpreted as a strategy for recruitment and expansion of muscle progenitor cells as supported by the cell culture experiments. Thus the CD34^+^/CD31^+^/VE-cadherin^+^/Flk-1^+^ cells posses the capacity to activate diverse genetic programs in response to peculiar environmental stimulation.

Interestingly, VEGF signalling seems to play the same role in vertebrates, modulating the migration, proliferation and survival of both endothelial and myogenic precursors cells into muscle regenerating areas [36], [37].

Taken together, these results suggest that: i) the hematopoietic/myoendothelial MyoD^+^/Flk-1^+^, MyoD^+^/VE-Cadherin^+^, MyoD^+^/CD34^+^ cells have the ability to directly participate in myogenesis both *in vivo* and *in vitro*; ii) VEGF secretion, while promoting stem cell recruitment and expansion, does not seem to be involved in inducing myogenic differentiation of myoendothelial cells; iii) leech hematopoietic and endothelial precursor cells share with vertebrates the same morphofunctional and molecular mechanisms, indicating that leeches can be considered a simple model for studying the regulating myoendothelial cell biology step by step.

## Materials and Methods

### Animals and Treatments

Leeches (*Hirudo medicinalis*, Annelida, Hirudinea, from Ricarimpex, Eysines, France) measuring 10×1 cm were kept in water at 19–20°C in aerated tanks. Animals were fed weekly with calf blood. Animals were randomly divided into separate experimental groups according to different protocols and treatments. Each treatment was performed at the level of the 80^th^ superficial metamere. Before each experiment, leeches were anaesthetized with a 10% ethanol solution.

Group (1): 5 untreated leeches that served as controls.

Group (2): 5 injured leeches; the animals were subjected to lesions consisting of a tissue explant (2×2×2 mm) affecting the entire body wall. The tissue explant was surgically removed with microdissecting scissors.

Group (3): 10 injured animals injected with the vital marker specific for endothelial and macrophages cells Dil-Ac-LDL (Acetylated Low Density Lipoprotein, Biomedical Technologies Inc., MA, USA). To functionally characterize the cells migrating into the injured area, animals were injected with 10 µl of a Dil-Ac-LDL solution (10 µg/ml in phosphate buffer (PBS) and after 4 h (corresponding to a suitable time necessary for the colorant to diffuse in the body wall of the animal, [20] the leeches were surgically wounded in the same region.

Group (4): 10 vascular endothelial factor (VEGF) injected animals. To functionally characterize the cells migrating under the influence of VEGF, the leeches were injected with the vital dye Dil-Ac-LDL (as above), and after 4 hours with 300 µl of PBS containing 100 ng/ml of VEGF_165_ (Pepro Tech, London, UK) in the same area.

Group (5): 10 untreated leeches injected only with Dil-Ac-LDL solution, as controls for both injured/Dil-Ac-LDL injected and to VEGF/Dil-Ac-LDL injected animals.

Group (6): 10 animals injected with Dil-Ac-LDL and Matrigel (MG) supplemented with VEGF. To selectively isolate the cells migrating under the influence of VEGF_165_, the leeches were injected with 10 µl of Dil-Ac-LDL (10 µg/ml in PBS buffer 0.01 M) and after 4 h were inoculated in the same area with 300 µl of MG (an extract of the murine Engelbreth-Holm-Swarm (EHS) tumor produced as previously described [38]) supplemented with 100 ng/ml of VEGF_165_, as previously described [20].

Group (7): 10 control matrigel injected animanls. The healthy leeches were injected only with liquid Matrigel (MG) that was not supplemented with the cytokine.

The animals were sacrificed after either 1 week, 15 days or 1 month from treatment, the stimulated areas or the MG implants were removed from the animal and processed for electron microscopy or embedded in Polyfreeze tissue freezing medium (Polysciences, Eppelheim, Germany) and immediately frozen in liquid nitrogen.

Cryosections (7 µm) obtained with a Leica CM 1850 were stained by differential staining (May Grunwald Giemsa, Bio Optica, Milano, Italy) or by immunocytochemistry to identify myoendothelial cells. Dil-Ac-LDL uptake was visualized on a fluorescence microscope Olympus BH2 through a rhodamine filter set (excitation/emission filters 550/580 nm). Images were acquired with a DS-5M-L1 Nikon digital camera system.

### Trasmission Electron Microscopy (TEM)

Samples were fixed for 2 h in 0.1 M cacodylate buffer pH 7.2, containing 2% glutaraldehyde. Specimens were then washed in the same buffer and postfixed for 2 h with 1% osmic acid in cacodylate buffer, pH 7.2. After standard serial ethanol dehydration, specimens were embedded in an Epon-Araldite 812 mixture. Sections were obtained with a Reichert Ultracut S ultratome (Leica, Wien, Austria). (Olympus, Tokyo, Japan). Thin sections were stained by uranyl acetate and lead citrate and observed with a Jeol 1010 EX electron microscope (Jeol, Tokyo, Japan).

### Indirect Immunofluorescence Staining

Cryosections of healthy, injured, Dil-Ac-LDL and VEGF injected leeches and cultured cells (the latter fixed with 4% paraformaldehyde in PBS for 30 min at 4°C) were pre-incubated for 30 min with phosphate-buffer (PBS) containing 2% bovine serum albumin (BSA) before the primary antibody incubation (4°C over night). The primary antibodies used were: mouse anti-human CD31, CD34, Flk-1 and VE-cadherin (1∶20, Santa Cruz Biotechnology, CA, USA); which reacts with leech endothelial cells, as previously demonstrated [14], [15], [17], [25]), mouse anti-human CD14 (1∶20, Santa Cruz Biotechnology), mouse anti-human CD68 (1∶20, DBA, Segrate, MI, Italy) and CD11c (1∶20, Novocastra laboratories, Newcastle upon Tyne UK) (which react with leech macrophages, as previously demonstrated [17], [25]), rabbit anti-human desmin (1∶100, Sigma, St. Louis, MO) which reacts with leech obliquely striated muscles fibres, as previously demonstrated [39], mouse anti-human A4.1025 (1∶10, Santa Cruz Biotechnology) which recognises many (if not all) sarcomeric skeletal myosin (MyHCs) in species from *Drosophila* to human and a rabbit anti-mouse MyoD (1∶20, Santa Cruz Biotechnology). The washed specimens were incubated for 1 h at room temperature with the appropriate secondary antibody (Jackson, Immuno Research Laboratories, West Grove, PA, USA) that was Cy3 or FITC (fluorescein isothiocyanate) conjugated (dilution 1∶100). In double labelling experiments, the primary antibodies mouse anti-human CD31 or CD34 or Flk-1 or VE-cadherin or CD14 or CD68 or CD11c, were incubated together with the primary antibody rabbit anti-mouse MyoD. After washing, the secondary antibodies donkey anti-mouse Cy3 conjugated and donkey anti-rabbit FITC conjugated were applied together on the sections. In cultured cells double staining with mouse anti-human CD34 and mouse anti-human A4.1025 was performed as described: anti CD34 was applied first, then cells were incubated with the secondary antibody goat anti-mouse FITC conjugated. After washing the cells were incubated with the antibody anti A4.1025 and subsequently, with the secondary Cy3 conjugated goat anti-mouse antibody. Nuclei were stained by incubating for 15 min with 4′,6-Diamidino-2-Phenylindole (DAPI, 100 ng/ml in PBS, Sigma). The PBS buffer used for the washing steps and antibody dilutions contained 2% BSA. Slides were mounted in Citifluor (Citifluor Ltd, London, UK) with coverslips and examined with fluorescence microscope Olympus BH2 (Olympus, Tokyo, Japan). The staining was visualized using excitation/emission filters 550/580 nm for Cy3, 490/525 nm for FITC and 340/488 nm for DAPI. Data were recorded with a DS-5M-L1 digital camera system (Nikon, Tokyo, Japan). Images were combined with Adobe Photoshop (Adobe Systems, Inc.). In control samples, primary antibodies were omitted and sections, pre-incubated for 30 min with PBS/BSA, were incubated only with the secondary antibodies. In the double labelling experiments the two types of secondary antibodies were applied together.

### Biochemical Procedures

The body walls of leeches were dissected into small pieces and homogenised in liquid nitrogen with a mortar. Rat skeletal muscle, used as a positive control, was homogenised following the same protocol. For SDS-polyacrylamide gel electrophoresis (SDS-PAGE), homogenates were suspended in extraction buffer (0.4 M NaCl, 5 mM MgCl_2_,1 mM EDTA, 0.5 µg/ml pepstatin, 50 mM Tris HCl, pH 7.2 with 1 mM phenylmethylsulphonyl fluoride and 0.2 mM ATP freshly added); particulate material was removed by centrifugation at 13000 rpm for 10 min at 4°C in a refrigerated Sorvall RCM14 microcentrifuge. Supernatants were denatured in 2X sample buffer (2% SDS, 10% β-mercaptoethanol, 0.002% bromophenol blue, 20% glycerol, 0.02 M Tris HCl pH 6.8) at 100°C for 5 min.

### SDS-PAGE

Analytical SDS-PAGE using 15% (for MyoD detection), 5% (for skeletal myosin detection) and 7.5% (for CD markers detection) acrylamide mini gel was made according to [40]. Molecular weights were determined by concurrently running, for the 15% mini gel, the Prestained SDS-PAGE Standard Broad Range (BIO-RAD, Hercules, CA) and, for the 5% and 7.5% min gel, the Novex Sharp Prestained Standard (Invitrogen, San Giuliano Milanese, MI, Italy). Electrophoresis was made at 200 V for 1 h.

### Western Blot

Proteins separated by SDS-PAGE were transferred onto Bio-Rad nitrocellulose filters according to [41]. Before immunostaining, the membranes were saturated with 5% BSA in Tris-buffered saline (TBS, 20 mM Tris-HCl buffer, 500 mM NaCl pH 7.5) at room temperature for 2 h. Nitrocellulose membranes were incubated overnight at 4°C with the primary antibodies: rabbit anti-mouse MyoD (Santa Cruz Biotechnology) or mouse anti-human A4.1025 (Santa Cruz Biotechnology), at a 1∶250 dilution or with the mouse anti-human CD34, CD31, antibodies (Santa Cruz Biotechnology) at a 1∶50 dilution in 2% TBS-BSA. After three washes of the membrane with TBS, antigens were revealed with peroxidase-conjugated secondary antibodies (1∶500): a donkey anti-rabbit for MyoD, (Jackson ImmunoResearch Laboratories) and a donkey anti-mouse for CD 31, CD34 and A4.1025 (Jackson ImmunoResearch Laboratories). After washing with TBS, signal was developed with chemiluminescent substrate (Pierce, Rockford, IL, USA). Stainings without the primary antibodies were performed as controls.

### Cultured Cells and *In Vitro* Assays

Ten leeches were injected at the level of the 80^th^ superficial metamere with 300 µl of Matrigel (MG) added with100 ng/ml VEGF_165_. After 1 week Matrigel implants were harvested from the animals, minced in small pieces using sterilized razor blades and mechanically dissociated with a micropipette in 400 µl of tissue culture medium [20]. To obtain single cell colonies for clonal analyses, cells were plated out at a low density (20/cm^2^) for 24 hrs and maintained at 20°C as previously described [20]. The distinct and well separated colonies of 1–3 cells that formed were then individually aspirated with a pulled glass capillary connected to a microinjector (Nanoject 2000, WPI, Germany), transferred to a single well of 96 well plates in 200 µl of tissue culture medium. After additional 72 hrs (3 days *in vitro*) some colonies were processed for histological and immunocytochemical analysis, while others were passaged into 24 wells plates and grown for additional 5 days (8 days *in vitro*) or 17 days (20 days *in vitro*) and processed as above.

To evaluate the effect of VEGF_165_ on cell proliferation and differentiation, colonies formed from single cells isolated from MG implanted 1 week as described above, were plated and expanded in 24-multiwell plates in medium alone or in medium supplemented with 100 ng/ml of VEGF_165._ All cultures were performed in quadruplicate and scored at 20 days in vitro using an inverted Olympus microscope. Data were recorded with a DS-5M-L1 digital camera system (Nikon).

### Assessment of Cell Viability with Trypan Blue

Cell viability was assessed using the vital dye Trypan blue, which is incorporated only in dying or dead cells. The cells were incubated at room temperature for 5 min with 0.4% trypan blue solution (diluted 1∶1 in basal medium) and directly observed using an inverted optical microscope (Olympus). Data were recorded with a DS-5M-L1 digital camera system (Nikon) within three minutes after from staining.

### Proliferation Assay

Cells, according to manufacturer's protocol (Amersham-Pharmacia, Buckinghamshire, UK), were incubated at 20°C for 1 h with the labeling medium (aqueous solution of 5-bromo-2′-deoxyuridine (BrdU) and 5-fluoro-2′-deoxyuridine 10∶1), washed in PBS and fixed in acid-ethanol (90% ethanol, 5% acetic acid, 5% water) for 30 min. After washing in PBS, cell proliferation was monitored by using a monoclonal mouse anti-5-bromo-2′-deoxyuridine and a secondary antibody anti-mouse IgG_2a_ HRP-conjugated to detect BrdU incorporation into cellular DNA. Signal was developed with a DAB solution and cells were examined with Olympus BH2 microscope (Olympus). Controls were performed by omitting the primary antibody.

### Statistical Analysis

In each section of four independent experiments the percentage of rounded cells co-expressing endothelial markers and the myogenic marker MyoD was calculated as follows: the total number of rounded cells stained by endothelial markers and the total number of cells co-expressing the endothelial and myogenic marker were counted by hand, using the Image J software package, (http://rsbweb.nih.gov/ij/download.html). The value of cells co-expressing the myogenic and endothelial marker was divided by the value of total number of cells expressing the endothelial markers.

For proliferation studies *in vitro*, cells belonging to single colonies were re-plated at a very low density of 20/cm^2^. Control cultures VEGF-treated cultures were counted after 20 days and the mean proliferation index has been computed by the ratio between the number of cells recorded after 20 days with the number of cells plated at day zero.

For the differentiation test, the total surface of the cultured wells was sampled in 30 fields of 87.000 µm^2^ each. Cells in each field were counted using the Image J software package and classified into round or elongated class according to their phenotype. For each field, the percentage of elongated cells on the total cell count has been calculated. Data represent the mean±SEM of the single field percentages. Statistical significance was assessed by an unpaired Student's *t* test using Origin 5.0 software (Microcal).
